# Hyper-acute EEG alterations predict functional and morphological outcomes in thrombolysis-treated ischemic stroke: a wireless EEG study

**DOI:** 10.1007/s11517-020-02280-z

**Published:** 2020-12-04

**Authors:** Miloš Ajčević, Giovanni Furlanis, Marcello Naccarato, Aleksandar Miladinović, Alex Buoite Stella, Paola Caruso, Tommaso Cillotto, Agostino Accardo, Paolo Manganotti

**Affiliations:** 1 Department of Engineering and Architecture, University of Trieste, Via A. Valerio, 10, 34127 Trieste, Italy; 2 Clinical Unit of Neurology, Department of Medicine, Surgery and Health Sciences, Cattinara University Hospital ASUGI, University of Trieste, Strada di Fiume, 447, 34149 Trieste, Italy

**Keywords:** EEG, NIHSS, Hyperacute ischemic stroke, Biomedical signal processing, Outcome prediction

## Abstract

Owing to the large inter-subject variability, early post-stroke prognosis is challenging, and objective biomarkers that can provide further prognostic information are still needed. The relation between quantitative EEG parameters in pre-thrombolysis hyper-acute phase and outcomes has still to be investigated. Hence, possible correlations between early EEG biomarkers, measured on bedside wireless EEG, and short-term/long-term functional and morphological outcomes were investigated in thrombolysis-treated strokes. EEG with a wireless device was performed in 20 patients with hyper-acute (< 4.5 h from onset) anterior ischemic stroke before reperfusion treatment. The correlations between outcome parameters (i.e., 7-day/12-month National Institutes of Health Stroke Scale NIHSS, 12-month modified Rankin Scale mRS, final infarct volume) and the pre-treatment EEG parameters were studied. Relative delta power and alpha power, delta/alpha (DAR), and (delta+theta)/(alpha+beta) (DTABR) ratios significantly correlated with NIHSS 7-day (rho = 0.80, − 0.81, 0.76, 0.75, respectively) and NIHSS 12-month (0.73, − 0.78, 0.74, 0.73, respectively), as well as with final infarct volume (0.75, − 0.70, 0.78, 0.62, respectively). A good outcome in terms of mRS ≤ 2 at 12 months was associated with DAR parameter (*p* = 0.008). The neurophysiological biomarkers obtained by non-invasive and portable technique as wireless EEG in the early pre-treatment phase may contribute as objective parameters to the short/long-term outcome prediction pivotal to better establish the treatment strategies.

Graphical abstract

**Figure:**
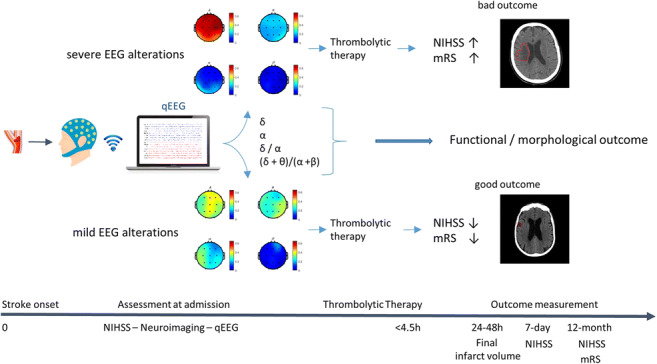
Block diagram of study protocol and main findings. Assessment at admission including wireless EEG acquisition in emergency setting (< 4.5 from stroke onset), extracted EEG features before reperfusion thrombolytic treatment. The main findings in our study sample are summarized in two different exemplificative stroke patients with different pre-thrombolysis alterations of EEG parameters resulting in different final infarct volume extensions and short/long-term clinical outcomes (NIHSS, mRS).

Block diagram of study protocol and main findings. Assessment at admission including wireless EEG acquisition in emergency setting (< 4.5 from stroke onset), extracted EEG features before reperfusion thrombolytic treatment. The main findings in our study sample are summarized in two different exemplificative stroke patients with different pre-thrombolysis alterations of EEG parameters resulting in different final infarct volume extensions and short/long-term clinical outcomes (NIHSS, mRS).

## Introduction

Ischemic stroke is a neuroemergency condition in which reperfusion therapy in selected patients can restore cerebral blood flow and lead to improvement or resolution of neurological deficits [24]. Thrombolytic therapy delivered within 4.5 h from stroke onset significantly improves the overall odds of a good stroke outcome [27]. Recent studies highlighted the efficacy and safety of thrombolytic treatment in extended time window, too (4.5–9 h from stroke onset or wake-up stroke) in patients selected by advanced neuroimaging [7, 8, 15, 20].

Early post-stroke prognosis is essential to better establish the treatment and rehabilitation strategies to improve recovery and minimize disability [6].

In the past, several independent early predictors of treatment-associated stroke outcome like age, sex, mean arterial pressure, history of diabetes, baseline glucose levels, baseline National Institutes of Health Stroke Scale (NIHSS) score, neuroimaging findings, time to treatment and recanalization, current smoking, and atrial fibrillation were reported [11, 24, 28].

However, early prediction of post-stroke outcome is still challenging since there is large inter-subject variability [31]. For this reason, reliable, non-invasive, inexpensive biomarkers that provide further prognostic information are still needed.

There is growing evidence concerning neurovascular coupling in the acute phase of ischemic stroke, thus neurophysiological biomarkers seem increasingly prominent to predict the outcome [29].

EEG, as a non-invasive neurophysiological biomarker, provides a rapid evaluation of instantaneous brain function due to its high temporal resolution. Nowadays, thanks to the new wireless solutions, this diagnostic technique can also be applied bedside in emergency settings, as is acute stroke management [32].

EEG changes in sub-acute ischemic stroke and their predictive power have been studied previously [6, 12–14, 37]. Abnormal EEG and generalized, but not focal, slowing were associated with clinical deterioration with an increase of NIHSS ≥ 3 from admission to discharge [37]. Furthermore, relative delta, alpha power, delta/alpha ratio (DAR), and the (delta + theta)/(alpha + beta) ratio (DTABR) EEG-related parameters have been associated with stroke functional and clinical outcome [12]. The relation between quantitative spectral EEG parameters in pre-thrombolysis hyper-acute phase and clinical and morphological outcome has not been studied yet. The thrombolysis currently represents the most common and efficacious treatment for intracranial vessel thrombosis. The study of the EEG alterations present before the treatment may contribute to the prediction of specific treatment outcome.

The aim of this study was to investigate the relation between early stroke-related EEG parameters, measured bedside with wireless EEG before the thrombolysis treatment, and short-term/long-term neurological disability measured with NIHSS and mRS in thrombolysis-treated ischemic stroke patients. In addition, we investigated the relation between quantitative EEG features with morphological outcome in terms of final ischemic lesion volume.

## Materials and methods

### Study population and protocol

The study included twenty patients (8 M/12F, mean age 75.8 ± 12.1 years) with first hyper-acute anterior ischemic stroke, who underwent EEG recording at bedside within 4.5 h since stroke onset before the reperfusion treatment. EEG recording was performed in the timespan between standard neuroimaging assessment (non-enhanced CT–NECT, Angio-CT, CT Perfusion–CTP) and the decision of possible reperfusion treatment. The wireless pre-set EEG system allowed hyper-acute bedside EEG assessment in emergency settings without compromising regular patient management and treatment. After the assessment, if the treatment inclusion criteria were fulfilled, patients underwent thrombolysis. Therefore, inclusion criteria were first-ever anterior circulation ischemic stroke undergoing systemic thrombolysis, in which EEG recording did not delay treatment decision and administration. Exclusion criteria were unknown stroke onset, wake-up stroke, stroke-mimic, previous stroke, hematic effusion, history of epileptic seizure, pre-morbid dementia, and use of medication such as neuroleptic or benzodiazepines were also exclusion criteria due to their effect on EEG assessment. The focus of the study was on thrombolysis-treated patients; therefore, patients who also received endovascular treatment were excluded.

The thrombolysis was performed with intravenous recombinant tissue plasminogen activator (rtPA) (0.9 mg/kg of body weight, maximum of 90 mg, infused over 60 min with 10% of the total dose administered as an initial intravenous bolus over 1 min).

During hospitalization, all patients received a standard clinical and diagnostic work-up, including a 22–36 h follow-up CT and neurological assessment.

The following data of included patients were collected: (1) demographic details (age, sex); (2) admission, 7-day, and 12-month (after stroke event) NIHSS scores; (3) premorbid and 12-month mRS; (4) stroke risk factors (hypertension, diabetes mellitus, dyslipidemia, smoking, obesity, ischemic cardiopathy, atrial fibrillation); (5) NECT (ASPECTscore); (6) lesion side; (7) symptomatic intracerebral hemorrhage (sICH) according to the definition of ECASS (European-Australian Cooperative Acute Stroke Study 3) [17]; (8) stroke etiology by TOAST classification [1]; (9) stroke syndrome by Bamford classification; (10) time from symptom onset to EEG assessment.

This study was approved by the Local Ethics Committee CEUR (Comitato Etico Unico Regionale, FVG, Italy) with approval number 115/2018. The research was conducted according to the principles of the Declaration of Helsinki. All participants released their informed consent.

### EEG acquisition and processing

EEG was acquired at bedside before the thrombolysis treatment and within 4.5 h from stroke onset by using Be Plus LTM amplifier @64 channels Wi-Fi (EBNeuro, Florence, Italy) and a wireless headset (EBNeuro, Florence, Italy) with 19 Ag/AgCl electrodes positioned according 10-20 system (Fp1, Fp2, F7, F3, Fz, F4, F8, T3, C3, Cz, C4, T4, T5, P3, Pz, P4, T6, O1, O2). All electrode impedances were kept below 5 kΩ, and sampling rate was set to 128 Hz. The offline analysis was performed using scripts developed in MATLAB (MathWorks Inc., Natick, MA). The signals were digitally filtered with the 0.5–40 Hz 2nd order Butterworth bandpass filter. For each of 19 unipolar channels, power spectral density (PSD) was estimated on 120 s artifact-free segments by using Welch’s periodogram [35], averaged on tracts of 10 s each, with applied Hann window. Subsequently, quantitative spectral parameters relative delta, theta, alpha, and beta power, as well as (delta+theta)/(alpha+beta) power ratio (DTABR) and delta/alpha power ratio (DAR), were calculated. The absolute power for each of spectral band (delta 1–4 Hz; theta 4–8 Hz; alpha 8–13 Hz; beta 13–30 Hz) was calculated for each channel, and then normalized with a total power across the 1–30 Hz range to obtain relative powers. In addition, also aforementioned DAR and DTABR were extracted. Relative power for each band, DAR, and DTABR parameters were averaged over all nineteen electrodes.

### Neuroimaging assessment

All CT imaging (NECT, CTA, and CTP) was performed with 256-slice CT scanner (Brilliance iCT; Philips Medical Systems, Best, Netherlands). In particular, CTP acquisition protocol involved intravenous injection of 75 ml of contrast medium, followed by a 40 ml of saline bolus, both administered at an injection rate of 4 ml/s. The exposure parameters adopted were 80 kVp and 150–200 mAs. Three-dimensional axial acquisitions on a whole brain volume with a reconstruction of the slices set to 5 mm were performed. The acquisitions were carried out every 4 s, resulting in a total scanning time of 60 s. CTP source image processing was performed, and CTP maps were calculated, as previously described [2, 16, 32]. Ischemic core and penumbra areas were identified by application of specific thresholds [36].

### Outcome measures

We have measured the clinical outcome in terms of NIHSS on the 7th day or earlier in case of discharge (7-day NIHSS) and 12 months after the ischemic event (12-month NIHSS). The NIHSS, consisting of 11 items to assess the main neurological functions, is the most adopted tool in the actual medical practice to evaluate stroke-related neurologic impairment at admission and evaluate clinical evolution and final outcome [1]. In addition, functional outcome was measured by modified Rankin scale (mRS) 1 year after the stroke event [34]. The mRS, an ordinal scale with 7 categories ranging from zero (no symptoms) to 6 (death), was the outcome measure: 0—no symptoms at all; 1—no significant disability despite symptoms, able to carry out all usual duties and activities; 2—slight disability, unable to carry out all previous activities, but able to look after own affairs without assistance; 3—moderate disability, requiring some help, but able to walk without assistance; 4—moderately severe disability, unable to walk and attend to bodily needs without assistance; 5—severe disability, bedridden, incontinent, and requiring constant nursing care and attention; 6—dead. Good outcome class was defined with mRS ≤ 2, while bad outcome class with mRS > 3.

Morphological outcome was evaluated in terms of final ischemic volume, which was identified by segmentation on follow-up NECT by using a seed-based region growing algorithm implemented in Matlab (MathWorks Inc., Natick, MA) and additionally manually outlined by two trained neurologists.

### Statistical analysis

The Spearman correlation was used to determine the degree of correlation between the EEG parameters and outcome measures (i.e., 7-day and 12-month NIHSS, final infarct volume). Statistical significance was set at *p* < 0.05. In testing the significance of correlation coefficients, as used for similar purposes in previous EEG studies [13, 14], the stringent Bonferroni correction for multiple comparisons was applied to maintain the total type I error rate at a sufficiently low level [18]. Univariate binary logistic regression was performed to assess the association between mRS ≤ 2 at 12-month and EEG parameters in their natural logarithm (ln).

## Results

Demographic, clinical, and neuroimaging data are summarized in Table 1. Mean age was 75.8 ± 12.1 years; median NIHSS at admission was 10 (range 3–23). Median time between onset of symptoms and hyper-acute EEG recording was 196 min (range 81–267 min). CT assessment at admission showed median ASPECT score of 9.5 (range 7–10).

**Table 1:** Participants’ demographics and clinical and radiological characteristics

Personal characteristics	*n* = 20
Age (years)	75.8 ± 12.1
Sex F/M	12/8
BMI	24.5 ± 2.8
Symptom onset–EEG (min)	196 (81–267)
ASPECTS	9.5 (7–10)
NIHSS at admission	10 (3–23)
NIHSS 7-day	4 (0–42)
NIHSS 12-month	2 (0–42)
mRS 0–2 *n*(%) anamnestic	18 (90%)
mRS 0–2 *n*(%) discharge	8 (40%)
mRS 0–2 *n*(%) 12-month	11 (55%)
Lesion side of the lesion L/R (*n*)	12/8
Bamford stroke subtypes	
TACI	6 (30%)
PACI	13 (65%)
LACI	1 (5%)
TOAST classification	
Atherothrombotic	4 (20%)
Lacunar	1 (5%)
Cardioembolic	8 (40%)
Cryptogenic	6 (30%)
Other cause	1 (5%)
CTP parameters	
Total hypoperfused tissue [ml]	61.0 (2.0–238.0)
Core (ml)	5.5 (0–86.0)
Mismatch	0.89 (0.32–1.0)
Final infarct volume(ml)	4.6 (0–116.5)
HTN (*n* (%))	14 (70%)
DM (*n* (%))	12 (60%)
Dyslipidemia (*n* (%))	15 (75%)
AF (*n* (%))	8 (40%)
ICM (*n* (%))	4 (20%)

Notes: participants’ reported age (year), sex (*n*), BMI (body mass index), Symptom onset—EEG assessment (min), ASPECTS, NIHSS at admission, NIHSS 7-day, NIHSS 12-month, anamnestic mRS < 3 (%), mRS at discharge < 3 (%), mRS at 12 months < 3 (%), lesion side (*n*), Bamford stroke subtypes (%) (total anterior circulation infarct, TACI; partial anterior circulation infarct, PACI; lacunar stroke, LACI), TOAST classification (%), CT perfusion parameters (ml), final infarct volume on follow-up CT (ml), and history of hypertension (HTN, %), diabetes (DM, %), dyslipidemia (%), atrial fibrillation (AF, %), ischemic cardiomyopathy (ICM, %)

Median NIHSS at 7-day and 12-month were 4 (range 0–42) and 2 (range 0–42), respectively. Eleven patients (55%) presented mRS ≤ 2 at 12 months. Median final ischemic volume calculated on follow-up NECT was 4.6 ml (0–116.0 ml). No sICH was observed.

Relative power of theta, delta, alpha and beta EEG bands, DAR, and DTABR calculated ratios, as well as correlation with 7-day NIHSS, 12-month NIHSS, 12-month mRS, and final ischemic volume, are reported in Table 2.

**Table 2:** EEG indices and their correlation with functional/morphological outcome

EEG parameter		NIHSS			Final infarct volume	mRS ≤ 2
	Median (range)	Spearman’s *ρ* (*p* value)			Spearman’s ρ(*p* value)	OR (95%CI) (*p* value)
	Admission	Admission	7-day	12-month	24–48 h	12-month
Delta	0.41 (0.25–0.67)	*0.82 (p < 0.001)*	*0.80 (p = 0.001)*	*0.73 (p = 0.003)*	*0.75 (p < 0.001)*	0.005 (0.000–0.494) (*p* = 0.023)
Theta	0.21 (0.09–0.33)	0.09 (*p* = 0.738)	0.10 (*p* = 0.737)	0.14 (*p* = 0.638)	− 0.06 (*p* = 0.801)	1.170 (0.091–15.021) (*p* = 0.904)
Alpha	0.15 (0.06–0.38)	*−0.87 (p < 0.001)*	*−0.81 (p = 0.001)*	*−0.78 (p = 0.001)*	*− 0.70 (p < 0.001)*	129.938 (2931–5760.319) (*p* = 0.012)
Beta	0.13 (0.05–0.21)	−0.29 (*p* = 0.312)	−0.21 (*p* = 0.460)	−0.19 (*p* = 0.511)	0.26 (*p* = 0.301)	1.402 (0.152–12.943) (*p* = 0.766)
DAR	3.26 (1.24–7.35)	*0.86 (p < 0.001)*	*0.76 (p = 0.002)*	*0.74 (p = 0.003)*	*0.78 (p < 0.001)*	*0.023 (0.001–0.380) (p = 0.008)*
DTABR	2.45 (1.13–4.66)	*0.85 (p < 0.001)*	*0.75 (p = 0.002)*	*0.73 (p = 0.003)*	*0.62 (p = 0.005)*	0.018 (0.001–0.493) (*p* = 0.017)

Median (range) of calculated EEG spectral parameters. Correlation between extracted EEG spectral parameters and NIHSS (admission, 7-day, 12-month) and final infarct volume at follow-up NECT (24–48 h); logistic regression for good outcome (mRS 12-month ≤ 2) using the EEG variables transformed into the natural logarithm.

*Note. Relative Delta, Theta, Alpha and Beta power, DAR delta/alpha power ratio; DTABR (delta + theta)/(alpha + beta) power ratio; NIHSS* National Institutes of Health Stroke Scale score; *mRS* modified Rankin scale; *ρ* Spearman’s rho correlation coefficient; *OR* odds ratio; *CI* confidence interval; statistically significant correlations are highlighted in italics.

**Fig. 1:**
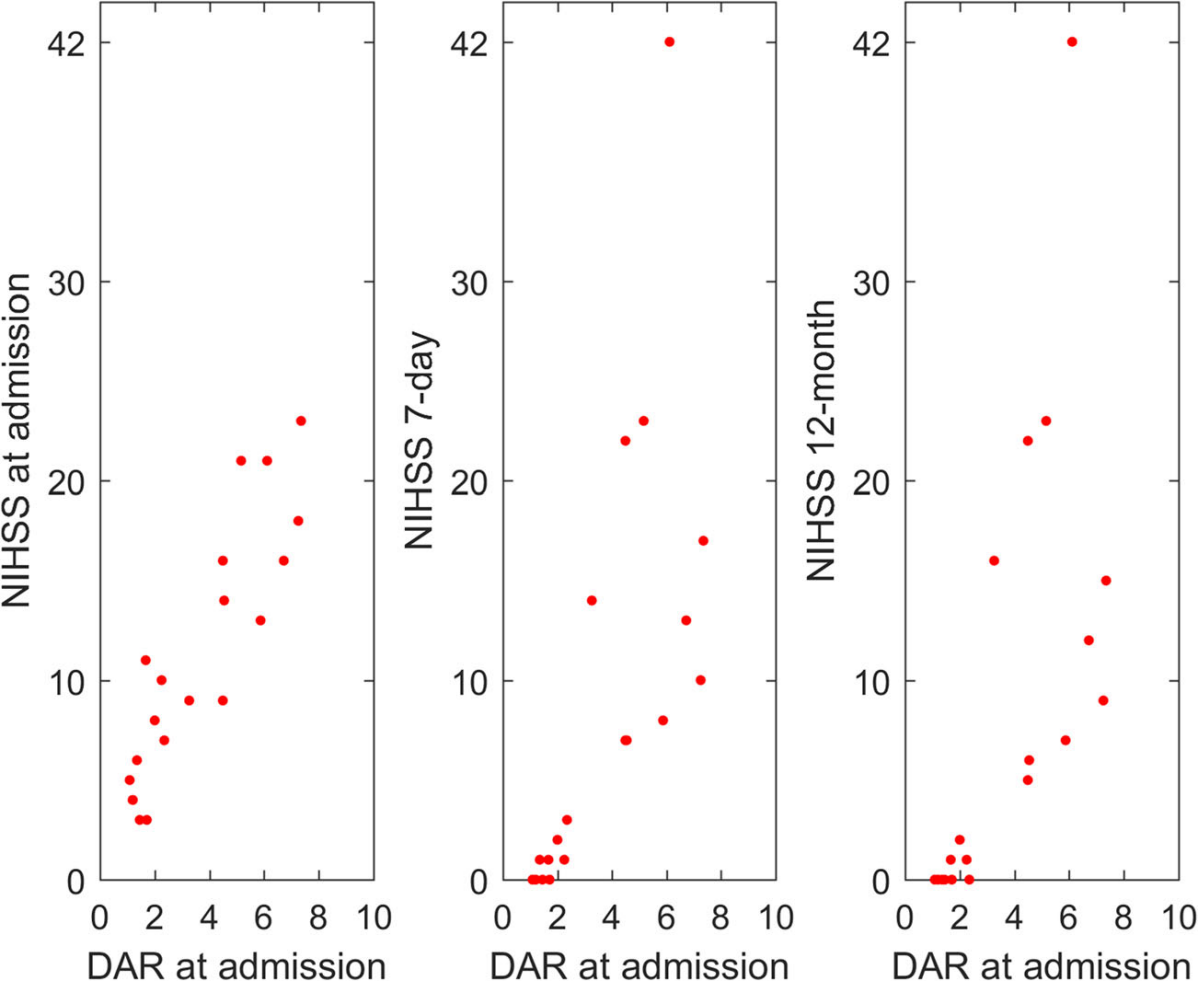
Neurological deficit in terms of NIHSS measured in the various time moments (admission, 7-day, 12-month) plotted against delta-to-alpha ratio (DAR) calculated from EEG tracings registered at admission. The correlation coefficients observed between DAR and NIHSS were rho = 0.86 (*p* < 0.001), rho = 0.76 (*p* = 0.002), and rho = 0.74 (*p* = 0.003) for NIHSS evaluated at admission, 7-day, and 12-month, respectively

Seven-day NIHSS as well as 12-month NIHSS correlated significantly with alpha (rho = − 81, *p* = 0.001, and rho = − 0.78, *p* = 0.001, respectively), delta (rho = 0.80, *p* = 0.001, and rho = 0.73, *p* = 0.003, respectively), DAR (rho = 0.76, *p* = 0.002 and rho = 0.74, *p* = 0.003; respectively), and DTABR (rho = 0.75, *p* = 0.002, and rho = 0.73, *p* = 0.003; respectively). The same EEG parameters correlated also with NIHSS at admission (Table 2). In Fig. 1, NIHSS measured in the various evaluation moments was plotted against DAR parameter calculated at admission.

A good outcome in terms of mRS ≤ 2 at 12 months was associated with DAR parameter (*p* = 0.008). The final infarct volume correlated with all EEG parameters, except theta and beta power. In particular, a strong direct correlation was observed for relative delta power, DAR, and DTABR (rho = 0.75, *p* < 0.001; rho = 0.78, *p* < 0.001; rho = 0.62, *p* = 0.005, respectively), as well as inverse for relative alpha power (rho = − 0.70, *p* < 0.001).

## Discussion

The study of the new early prognostic factors that modulate the stroke outcome may support a personalized therapeutic intervention to improve patients’ recovery. Neurophysiological biomarkers seem increasingly applicable to predict outcome [6, 12] owing to the growing evidence of neurovascular coupling in acute ischemic stroke [29, 32]. Early pre-treatment EEG parameters in the hyper-acute phase (< 4.5 h from symptom onset) have not yet been studied as predictive factors of the short- and long-term outcome in thrombolysis-treated stroke patients.

The main finding of this study is that early EEG parameters assessed bedside by wireless devices may contribute to the prediction of neurological deficit, functional disability, and morphological lesion at discharge and at 12 months in thrombolysis-treated stroke patients.

**Fig. 2:**
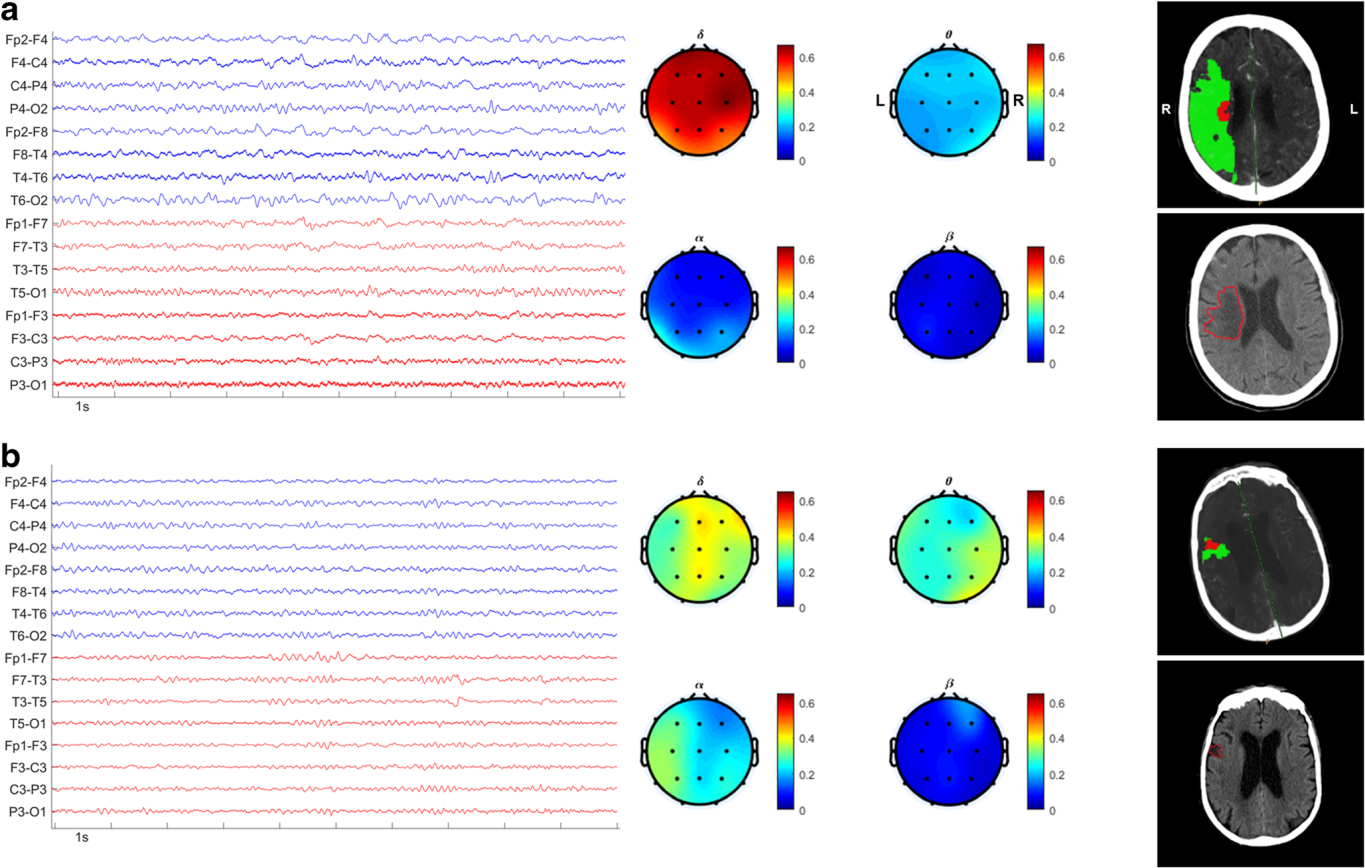
**a** Acute stroke patient with bad short/long-term functional and morphological outcomes. Baseline features: female, 79 years, premorbid mRS = 0, NIHSS at admission = 21, TACI, cardioembolic etiology, ASPECTS = 9, right M2-occlusion, CTP total hypoperfused volume = 149.6 ml, CTP core volume = 10 ml, relative spectral powers delta = 0.66, theta = 0.16, alpha = 0.13, beta = 0.05, DAR = 5.08, DTABR = 4.55. Clinical and morphological outcomes: NIHSS 7-day = 23, NIHSS 12-month = 23, mRS at discharge = 5, 12-month mRS = 5, final infarct volume = 73.3 ml. From left to right: 16-channel longitudinal bipolar montage of EEG raw data, EEG spectral power topographic maps, CTP core-penumbra summary map with estimated necrotic core area (MTT higher than 145% of the contralateral healthy area and CBV < 2.0 mL/100 g), and salvageable penumbra area (MTT higher than 145% of the contralateral healthy area and CBV > 2.0 mL/100 g), highlighted in red and green, respectively, follow-up NECT with delineated final infarct lesion. **b** Acute stroke patient with good short/long-term functional and morphological outcomes. Baseline features: male, 87 years, premorbid mRS = 1, NIHSS at admission = 6, PACI, cardioembolic etiology, ASPECTS = 10, right distal middle cerebral artery occlusion, CTP total hypoperfused volume = 8.5 ml, CTP core volume = 2.8 ml, relative spectral powers delta = 0.33, theta = 0.29, alpha = 0.23, beta = 0.15, DAR = 1.43, DTABR = 1.63. Clinical and morphological outcomes: NIHSS 7-day = 1, NIHSS 12-month = 0, mRS at discharge = 1, 12-month mRS = 1, final infarct volume = 3.6 ml. From left to right: 16-channel longitudinal bipolar montage of EEG raw data, EEG spectral power topographic maps, CTP core-penumbra summary map with estimated necrotic core area (MTT higher than 145% of the contralateral healthy area and CBV < 2.0 mL/100 g), and salvageable penumbra area (MTT higher than 145% of the contralateral healthy area and CBV > 2.0 mL/100 g), highlighted in red and green, respectively, follow-up NECT with delineated final infarct lesion

In particular, 7-day and 12-month NIHSS outcomes were inversely related to relative alpha power and directly related to relative delta power as well as DAR and DTABR parameters. A good clinical outcome measured with mRS ≤ 2 at 1 year was strongly associated with DAR. Moreover, the final infarct volume was significantly associated with all considered EEG parameters, except theta and beta. In Fig. 2 are reported two different exemplificative cases of patients admitted to our stroke unit with an acute anterior ischemic stroke with different pre-thrombolysis EEG alterations resulting in two different functional outcomes and final infarct volume.

These findings support the hypothesis that these objective biomarkers, obtained by non-invasive and portable technique as wireless EEG, may add important short- and long-term prognostic information in hyper-acute thrombolysis-treated stroke patients. Indeed, early neurophysiological alterations measured by the identified EEG parameters were associated both to the clinical/functional and morphological outcomes. The clinical/functional outcome is highly related to the final extent of the ischemic infarct, although this is also dependent on lesion location [5, 19, 23]. A preserved alpha power with limited increase of delta power in hyper-acute pre-treatment phase is indicative of neuronal survival in the ischemic regions and consequently of a good prognosis after the reperfusion treatment.

The still arduous stroke outcome early prognosis may also be improved by taking these EEG pre-treatment markers into account to reduce the gap due to large inter-subject variability. Recently, a sub-acute EEG stroke study showed that outcome models including quantitative EEG parameters and other clinical/neuroimaging stroke outcome predictors were found to be superior to models without EEG data [6].

The sub-acute stroke studies reported significant functional and clinical predictive power of relative delta, alpha power, DAR, and DTABR EEG-related parameters [6, 12, 14, 30]. Finnigan et al. reported that sub-acute (49 ± 3 h post symptom onset) DAR ratio (DAR; *r* = 0.91, *p* < 0.001) and relative alpha power (*r* = 0.82, *p* < 0.01) were significantly correlated with 30-day NIHSS score in thirteen ischemic stroke patients [14]. Sheorajpanday et al. found a significant correlation between EEG (acquired from 3 to 168 h since symptom onset, median 24 h) parameters and mRS at 6 month in 75 patients with a first ever stroke (0.42, *p* < 0.0005 for the pairwise derived Brain Symmetry Index (pdBSI) and 0.43, *p* < 0.0005 for DTABR) [30]. The same study observed a significant correlation between pdBSI and NIHSS 7-day and final infarct volume but not between other EEG parameters, in 21 patients with an early EEG registration in the timespan between 3 and 6 h since stroke onset [30]. Bentes et al. observed that delta, alpha, beta relative powers, and DTABR predicted outcome in terms of mRS on discharge and at 12 months in 151 anterior ischemic stroke patients recorded within 72 h. In this cohort, DTABR and alpha relative power had a higher discriminative predictive capacity [6].

However, the aforementioned report results were obtained in cohorts in which just a small portion of patients underwent reperfusion treatment and EEG was collected in post-acute and/or post-treatment phase. Our study is the first that investigated early pre-thrombolysis predictive power of EEG features. The obtained results in homogeneous cohort are important because of growing diffusion of reperfusion therapy [22, 27] also in time-extended cases [2, 4, 7, 8, 15, 20, 25], and may thus contribute to guiding treatment and rehabilitation strategies in the earliest phase of ischemic event in the era of personalized medicine.

Portable wireless device enabled our study to adopt early EEG recording, thus avoiding to delay the treatment and compromise the patient management. The wireless EEG device digitized analog EEG electrical signals at a point close to the electrodes and subsequently transmitted EEG signals via Wi-Fi protocol to a base station. This allowed to minimize movement of electrode wires, a major source of electromagnetic interference [33] and electrode displacement that dramatically degrades EEG signal quality. Our study also showed that adequate acceptable quality of EEG data can be obtained in such adverse recording conditions as stroke-emergency setting. The state-of-the-art portable technologies may overcome most of EEG application inhibiting factors occurring in emergency settings such as stroke units [9, 10].

Perfusion neuroimaging, such as MRI and CTP techniques, are crucial in ischemic stroke assessment and patient selection due the identification of ischemic core and salvageable hypoperfused penumbra [15, 16, 21, 26] However, these techniques are not feasible tools to monitor brain ischemia evolution in the acute phase. Brain oscillatory activity changes occurring in acute ischemic stroke are related to neurophysiological changes (measured by EEG) in the cerebral tissue during hypoperfusion as manifestation of neurovascular coupling [29, 32]. Hence, EEG in the hyperacute phase could be a feasible instrument of real-time functional monitoring, considering its high correlation also with neurological deficit at admission [3], as well the correlation in the later acute phases [14], can be potentially considered in the future as an objective surrogate parameter for functional monitoring.

This study has some limitations. Owing to non-ideal bedside acquisition conditions in the first minutes of hospitalization in an emergency setting, artifacts related to the initial medical assessments and nursing care could not be avoided. In addition, the results were obtained from a limited sample of a single Stroke Unit and should be confirmed in a larger study. Due to the limited sample, we performed only univariate analysis; further studies on broader sample are needed to identify the strongest EEG predictive factor. The aim of this preliminary study was to assess the informativeness of EEG objective instrumental parameters in the pre-treatment stage. Not only may EEG at admission deliver a static picture as does neuroimaging, but it may also offer a continuous monitoring of acute neuro-physiological changes over time, thus updating the information on patients’ clinical condition and prognosis. Despite the above-mentioned limitations, our cohort was a homogenous and representative sample of hyper-acute (median time from symptom onset to EEG assessment = 196 min) anterior circulation stroke patients, with a median NIHSS score of 10 (3–23) and a mean age of 75.85 ± 12.1 years.

## Conclusions

The results of this hyper-acute wireless EEG study showed that relative delta power, relative alpha power, DAR, and DTABR ratios correlate with neurological deficit and morphological lesion at discharge and at 12 months in thrombolysis-treated stroke patients. These neurophysiological biomarkers, obtained by non-invasive and portable technique as wireless EEG in the early pre-treatment phase, may contribute as objective parameters to the short- and long-term outcome prediction, which is pivotal to better establish the treatment strategies. The biomarkers may provide a bedside real-time picture of the probable response to thrombolytic therapy and may be potentially considered in the future as an objective surrogate parameter for longitudinal monitoring.
